# Proceedings of the Medical Convention

**Published:** 1871-07

**Authors:** 


### PROCEEDINGS OF THE MEDICAL CONVENTION.

We have been furnished, by the Secretary, Dr. Duncan, of Savannah, Ga., the proceedings of the late Medical Convention which was convened at Macon, Ga. This Convention was called by a large number of the leading and most respectable physicians of the State, who were opposed to the unparliamentary and unconstitutional proceedings of the Georgia Medical Association, which met at Americus, Ga., April last. We challenge any man to undertake to defend the Americus meeting as constitutionally conducted, or as truly, or fairly representing the sentiment of a vast majority of the profession of the State. Indeed the profession were not aware that an effort would be made by certain parties, by concentrating their entire strength, to surreptitiously get possession of the Association in order to carry out their purposes. Nor did they, on the other hand, suppose that a Convention for the purpose of condemning their action, would be packed in the same way—particularly as the leaders of this party had, by publication, give the callers to understand that *no one would attend the Convention but those who were opposed to the action taken by them at Americus*, in this way deceiving the profession as effectually as had been done by the same strategy before the meeting at Americus.

The Convention was called by over one hundred of the best medical men of the State. Very few of these attended—say about thirty-eight. The time was unpropitious for the assemblage of such a Convention, both as regards the high temperature of that month and the pressing business of the profession at that season of the year. The opinion, as already stated, prevailed, that it was not supposed that parties who had refused to sign the call and had condemned the Convention, would bend all their energies to have their last man on the ground, for the purpose of continuing the strife and prolonging a controversy they had thrust upon the profession. The proceedings will show the animus of this party, as well as the true object of the signers of the call. If this party is *afraid* to have an investigation and by a majority of one, adjourn a Convention in the face of a proposition, coming from a conservative gentlemen, to settle the difficulty—then the odium of continuing the strife and severing the profession must fall upon them. By the meanest of accidents, a few men—scarcely a Corporal's guard of the profession—have succeeded in placing themselves in power over twelve hundred professional men of Georgia. However unwise in the profession to allow it, still we are not disposed to lament its occurrance, as it reveals two very important facts—first that not forty men can be found in Georgia to meet to uphold the falling fortunes of a College which must be rescued from its perilous condition, or be numbered with the things that were. Second, that the profession have had demonstrated to them in the most effectual manner the objects and the means employed to carry them out, of a party at war with its interests, and subversive of its honor and integrity. For these reasons we are not disposed to complian, knowing that when the profession in its power and authority shall arise, it will shake off the incubus, and render up a judgment strictly in accordance to the demands of medical law, and in the interest of professional peace and harmony.

This much we feel bound to say—not from any "personal feeling," but as Journalists, to whom a large number of the profession look to be informed of events transpiring within our ranks. We have never, knowingly, deceived the profession. We shall continue to advocate and uphold principle, and denounce error. We do this for the sake of principle and the good of the profession. We cherish enmity toward none, but when they collide with the law, then the law, and not individals, must be blamed. Without law medical organizations must cease to exist, and the profession be ingulphed in ruin. Without the enforcement of law the profession will be wrecked, hopelessly, upon the rocks of charlatanism and quackery, and the professional man who, silently and unmoved, views principle after principle, and law after law broken, without being aroused to action, is as guilty of hurling the profession from its high pedestal as those who, with wicked hands sieze upon and overthrow it. They are neither the friends of the people nor the profession. To sustain and fearlessly advocate principle is the highest duty of man. To compromise it; to bid it step aside that falsehood, under the guize of *policy* may be exalted, can never accomplish honorable results or gain the approbation of good men.

Such efforts will always end disastrously to those by whom they are employed. The good man cannot compromise without yielding up a part of the truth, and error never yields until the last vestige of principle is lost. To compromise religiously is to open wide the door to infidelity. To compromise politically is to invite anarchy and ruin. Either will be but a question of time in working out their results. We have entreated our medical brethren to stand firmly to truth and principle. If they refuse to do it the next generation will see a prostituted calling, once professional, debauched and in the hands of ignorant pretenders and quacks. Nothing can save the profession but a firm, unyielding devotion to its principles and laws.

We have an abiding confidence in the honor of the profession. Believing this, and while deploring the existence of the controversy, so unfortunately thrust upon them, we ask in behalf of principle and truth, and those who have supported these, that the profession investigate the causes of it, that truth may triumph and principle be victorious.

